# Glasgow Royal Infirmary

**Published:** 1831-04

**Authors:** 


### GLASGOW ROYAL INFIRMARY.

*Cases of Traumatic Tetanus.* [From the Report of R. Perry, M.D., Senior Surgeon; *Glasgow Med. Journal.*]

The following cases of tetanus are chiefly interesting from the facts noticed on inspection, which, as far as I know, have not been hitherto attended to; and, if corroborated by the inspection of similar cases, will add another link to the chain of causes which lead to a correct pathology of a disease, the most painful to witness, which has hitherto rested wholly upon conjecture.

Case I. Patrick Vallily, ætat. fifteen. 17th April, 1830. A few hours ago while sitting near the funnel of a steam-boat engine, the boiler exploded, and he was lifted into the air. Both legs, and posterior part of left thigh, are extensively vesicated, both arms and shoulders slightly so, occasioned by the hot water thrown on him. Pulse quick and feeble; has had vomiting.—Sumat statim Tinct. Opii gtt. xl.

18th. Occasional vomiting; in other respects easy. Hab. Opii gr. i. vesp.

19th. Seems confused, but no return of vomiting; complains only of pains of abdomen, which is slightly tender on pressure. Tongue white and moist; pulse 100; bowels open.—Cont. Opii gr. i. vespere. Adhibeant. abdomini hirud. xii.

20th. A rigor this morning; half an hour after, was bled to nine ounces. Blood, first cup buffy. Complains of slight pain of abdomen on motion, but there is no tenderness on pressure. Pulse

110; tongue white; thirst; bowels slow.—Rep. Infus. Sennæ c. Sulph. Magnes. Vespere: rep. venesect.

21st. Bled to six ounces. Pulse 120; tongue less white; bowels open. Feels much easier.

26th. Since last bleeding, has continued much easier. Pulse has fallen in frequency, and tongue cleaning.

29th. Convalescent till 27th, when complained of pain in abdomen, not increased on pressure: had an opiate, which was repeated last night. Today was found lying on his back, head retracted, and muscles of the head and trunk rigid; countenance anxious, and features retracted. Slight difficulty on deglutition, but can open his mouth pretty freely. Pulse 105; tongue whitish at edges, brownish and dry in centre.—Sumat st. Calom. ℈i., et post hor. tres Infus. Sennæ donec plen. dejec. alv.; post. sol. alvi hab. enem. c. Tinct. Opii ʒi.; spin. applic. vesicat.

30th. Physic operated well, and in the evening less permanent rigidity of the muscles, but the accumulation of phlegm in trachea most annoying. Spasms increase in frequency; complains of pain in the region of the heart; pulse 124; tongue brown and dry in centre.—Sumat tertia q. q. h. Calom. gr. x. c. Opii gr. iss.; curet. pars vesicat. Ungt. Sabinæ.

May 1st. It was at this date the patient first came under my care. Since last report, the spasms of the muscles of the trunk have become more severe and permanent; less able to open his mouth; severe pain at epigastrium; no stool for the last twenty-four hours; has continued the calomel. To have powdered opium sprinkled on the vesicated portions of legs.

2d. Thirst urgent; tongue dry; pulse 110.—℞. Ol. Croton. gtt. iij.; Pulv. Sacc. gr. vi. M. et divid. in d. iij. sum. i. tertia q. q. h. Applic. Catap. com. vesicationibus. Omit. P. Opii.

3d. Has had several dark stools from the croton oil; tension, and pain of abdomen, diminished, but spasms still continue. Pulse in the morning feeble, but become stronger since taking a little wine.

Four P.M. Pulse sunk considerably; in consequence of which, four ounces of spirits were ordered to be taken in divided doses, with warm water and sugar.

Spasms continued till five o'clock next morning, when he died. The body was immediately placed with the face to the floor.

*Inspection, twenty-four hours after death.* The whole spinous processes and calvarium were removed, the brain and thecæ vertebrarum fully exposed. There was a little serous fluid at the base of the brain, betwixt the tunica arachnoidea and pia mater. The brain was considerably more vascular than usual, and on the posterior part of both lobes of the cerebellum there existed an ecchymosed appearance, which could easily be removed by raising the pia mater. The medulla spinalis had a perfectly healthy appearance, but a considerable quantity of partly fluid, partly coagulated blood, existed betwixt the theca and the vertebræ. The vesicated surfaces occupied the lower half of the left leg, and the outer and lower half of the right leg: both had a green sloughy aspect, and the cellular substance was much inflamed. The veins did not appear more vascular than natural, and the arteries appeared healthy. The nerves were also carefully examined; the cutaneous nerves of both legs, particularly the communicans tibialis and the communicating branches of the peroneal nerve with the tibialis communis, were inflamed at the seat of the injury; tracing them upwards above this point, they were perfectly healthy, except that portion of the peroneal which turns over the head of the fibula; there it was again distinctly very vascular, thus leaving an intermediate portion perfectly free from the appearances of inflammation. The vascularity appeared to be confined to the sheath of each nerve; the deep-seated branches appeared to be quite natural. No other morbid appearances were detected.

Case II. Wm. Fleming, ætat. seventeen. 22d July, 1830. Eight days ago, the ring and middle fingers of the right hand were drawn in betwixt two teethed wheels, and the integuments much lacerated; the last phalanx of the middle finger was completely crushed, and separated from the second, except at its fore part, where a small slip of skin kept it adherent; this was removed shortly after the accident, and the fingers dressed at first with adhesive straps; the day before admission, had poultices applied. Last night began to experience severe pain in fingers, which, before yesterday, had been tolerably easy: at the same time, was seized with tetanic symptoms, of stiffness of the muscles of the neck and lower jaw, and pain at epigastrium.

On admission today, at two P.M., the symptoms above related, somewhat aggravated, but did not prevent him walking up to the hospital: there is at present slight rigidity of the sterno-mastoid muscles; deglutition easy. The second and last phalanges of the injured fingers are completely gangrenous, and the integuments separated from the first, exposing the bone, of a black colour; has severe pain in bruised fingers, very much increased on the slightest pressure; pain does not stretch up arm. The bones of the second phalanx of both fingers are fractured; the fore and little fingers are uninjured. Bowels are easy. Had twelve grains of calomel immediately on admission, and fourteen leeches applied to the nape of the neck, and, at six P.M., both bruised fingers were removed. The middle finger was taken off at its junction with the metacarpal bone and the two last phalanges of the ring finger. Torsion of the arteries was used in place of ligatures, to stop the hemorrhage, (a practice I have always adopted in amputations of the fingers and toes,) during the operation, of which he complained much; had distinct opisthotonos. The calomel not having operated, was ordered Sulph. Magnes. ℥ij., Tart. Ant. gr. ¼, o. h.

23d. The salts and tartar emetic were continued every hour during the night. Bowels have been freely opened; vomited occasionally. Muscles of the back and belly have become rigid, and at times distinct opisthotonos occurs; is unable to open his jaws so far as to put out his tongue; the attempt to do so generally brings on general spasms. Complains much of pain of right breast; pulse 140, full and soft; skin moist; slight headach; makes water freely; has some difficulty in swallowing.—Cont. Tart. Ant. gr. ¼ tantum. o. h. Omitt. Sulph. Magnes. Hab. Acetat. Morph gr. ½ o. h.; Fricet. Pect. c. Tinct. Opii et Sap., et colli nuch. app. vesic.

Ten P.M. Spasms less frequent, but more severe; can open mouth better; has had no stool since visit at one P.M.; has taken regularly the quantity of morphia and tartar emetic prescribed; feels drowsy, and has vomited a little. Pulse 160, full, and rather hard; water has been drawn off by catheter. Repet. Sulph. Magn. et Tart. Antimon. ut antea.

24th. Died this morning at seven, the spasms continuing both frequent and severe.

*Inspection, twenty-four hours after death.* The body was allowed to lie the usual way, on the back, till the time of inspection. The calvarium and spinous ridges were removed, fully exposing the theca vertebrarum, down to the cauda equina; there was no effusion on the brain or its membranes, and its substance was natural throughout. No effusion existed between the theca and the vertebræ: the theca was healthy, and betwixt it and the spinal cord was a preternatural quantity of serum. The cord itself was of a pale colour. The nerves on each side of the remaining phalanx of the ring finger were very vascular. On tracing upwards the ulnar nerve from this point to the elbow, it was of its natural colour; but here again it became very vascular for about the extent of two inches. In the axilla it again presented a similar appearance as at the elbow, the portion of it intervening betwixt these two points being healthy. Tracing the median nerve in the same way as the ulnar, it was found perfectly natural, from its digital branch, which supplied the radial side of the ring finger, (and which, as stated above, was much inflamed,) till about the middle of the arm, when it again presented an inflamed appearance for the extent of an inch and a half. The portion of it intervening betwixt this part and that confined to the axilla, where it again became vascular, was natural. This vascularity throughout, was not confined to the sheaths of the nerves, but occupied their substance; the radial and superficial nerves of the arm, along with its veins and arteries, were perfectly natural; the lumbar nerves were unaffected; the œsophagus was examined, and found healthy; the trachea appeared inflamed, and contained a large quantity of greenish coloured mucus; the other thoracic viscera and digestive organs natural.

The plan of treatment followed in the above cases may be considered as purely empirical: indeed, the treatment of this disease may be said to have been hitherto uniformly so, and must continue so while the seat and nature of the disease is unknown, as remarked by Mr. Cooper, in his excellent Surgical Dictionary: "Nothing is a more certain proof of our not being acquainted with any very effectual method of treating a disease, than a multiplicity of remedies, which are as opposite as possible in their effects." To give even a summary of the remedies employed, and the plans of treatment strongly recommended, would occupy too large a space, and be of little use, from all of them being founded upon conjecture. Although the morbid appearances in the two inspections related correspond very closely, it would perhaps be rash to found upon them, until confirmed by other cases, any certain plan of treatment; yet I think I would be warranted in treating any case of the kind which might occur, as a local inflammation of the nerves leading from the seat of the injury, the interruption of the suppurative process in the wound being one of the first appearances. When the tetanic symptoms arise from fracture of any of the fingers or toes, or even compound or comminuted fracture of the larger extremities, we might be warranted in having recourse to amputation: at all events, a strict antiphlogistic treatment, with the application of numerous leeches in the course of the affected nerves, followed by blisters, ought not to be neglected; warm poultices, stimulating fomentations, or the turpentine liniment, ought to be applied to the wound, and these local remedies, accompanied with the free exhibition of emetic tartar, either combined with Sulph. Magnesiæ dissolved in water, or with calomel and opium in small but repeated doses, so as to act both on the skin and bowels: the torpid state of the latter in this disease, indicate an interruption or weakened state of the nervous system, which may arise from the increased expenditure or exhaustion of nervous power by the diseased parts.

